# The practical other: teleology and its development

**DOI:** 10.1080/03080188.2018.1453246

**Published:** 2018-04-26

**Authors:** Josef Perner, Beate Priewasser, Johannes Roessler

**Affiliations:** a Centre for Cognitive Neuroscience, University of Salzburg, Salzburg, Austria; b Department of Psychology, University of Salzburg, Salzburg, Austria; c Department of Philosophy, University of Warwick, Coventry, UK

**Keywords:** Theory of mind, teleology, theory theory, simulation theory, rationality theory, reasons for action, perspective taking, counterfactual reasoning

## Abstract

We argue for teleology as a description of the way in which we ordinarily understand others’ intentional actions. Teleology starts from the close resemblance between the reasoning involved in understanding others’ actions and one’s own practical reasoning involved in deciding what to do. We carve out teleology’s distinctive features more sharply by comparing it to its three main competitors: theory theory, simulation theory, and rationality theory. The plausibility of teleology as our way of understanding others is underlined by developmental data in its favour.

## 1 Introduction

In this paper, we are concerned with a single but central aspect of the way we make sense of the other: our grasp of intentional agency. We want to argue that the way we make intelligible what others intentionally do rests on the same processes by which we interpret our own actions. Our interpretations consist of the practical reasoning schema. We aim for a more attractive state of the world than it is presently in. This more desirable state is our goal. Then we take actions which will be instrumental in bringing about the goal. For a practical example, suppose you go to the cafeteria to have a bite to eat. A simple-minded way to make sense of your action would be to suggest that you are doing what you are doing for a good reason, viz. that it is desirable that you should have something to eat, and that going to the cafeteria is an effective, and perhaps, in the circumstances, the best, way for you to secure food. This simple schema has several noteworthy features:

(1) Teleology. The schema is teleological as the actions are directed at bringing about a goal state.
(2) Value facts. The goal is something ‘good’ in some minimal sense of being attractive, desirable, needed, worthwhile having, etc. This conforms to how Aristotle conceived of teleological explanations: the goal is ‘the good to be achieved’ (Charles 2012, 227).
(3) Normative reasons. That it is desirable that one should do x and that doing y is the best way for one to do x are considerations that, put together, *count in favour* of one’s doing y or, in other words, provide a normative reason for one’s doing y (Scanlon 1998). A normative reason can be used to justify an action or to bring out its *point*. That such reasons can properly be requested is definitive of intentional agency, as distinct from mere bodily movements (Anscombe 1957). (On the obligation to give reasons c.f. contribution by Buddeberg (2018))
(4) Objectivity. The reasons for acting are objective facts, not mental states representing, or purporting to represent, facts. It’s the fact that there is food to be got in the cafeteria and the fact that having something to eat would be good for me that, put together, constitute the reason I have for proceeding to the cafeteria. If it turns out the cafeteria is closed, that would mean you were wrong to think you had a good reason for going there.
(5) Publicity. Reasons for acting are not ‘private’ affairs but public. The fact that having food would be ‘good for you’ bears not just on *your* practical reasons but also, potentially, on mine: it may give me a reason to get food for you, or to facilitate your efforts to obtain food, or at least not to obstruct your attempt to do so.

Ordinary action understanding, on our analysis, assigns a central *explanatory* role to *normative* reasons – where such reasons are not mental states but combinations of evaluative facts and facts about the agent’s practical abilities. Does that mean mental states have no role to play in teleological explanations? At this point, we need to make a distinction (see Perner and Roessler 2010; Roessler and Perner 2013). ‘Pure teleology’ would make sense of what you are doing simply in terms of the worldly facts that constitute good reasons for your action, with no regard to your perspective on your reasons – without, for example, taking into consideration whether you are aware of those facts. Below, we will set out evidence in support of the developmental hypothesis that we start our social lives as ‘pure teleologists.’ Note, though, that even pure teleology may involve appeal to mental states. For example, certain kinds of mental states may be implicated in understanding the relevant evaluative facts. One thing that can make it desirable that you should eat something is that this would put paid to your unpleasant feeling of hunger. Note, also, that the agent’s *practical abilities* are to the fore in pure teleology. A pure teleologist needs to attend carefully not just to the situation in which an agent finds herself but also to the needs and skills she brings to the situation (on the role of empathy and theory of mind for prosocial behaviour and the overlap of brain regions involved, see Kanske (2018)).

Nevertheless, ‘pure teleology’ is limited and inadequate in certain important respects. We arrive at a more sophisticated style of teleological explanation – ‘teleology-in-perspective’ – by adding two kinds of complexity. One kind has to do with the conditions that must be fulfilled for normative reasons to ‘make a difference.’ Crudely: unless you are *aware* of the reason-giving facts, and recognize them as reasons, they can’t really be *your* reasons for doing anything and so are not likely to impact on your activities. Accordingly, teleology, in a first step towards ‘teleology-in-perspective,’ will give a central explanatory role to factive mental states (states that involve relations to worldly facts), such as knowing, recognizing, realizing, or perceiving something to be the case. Of course, even sophisticated teleologists will often simply invoke the normative reasons for which someone is acting, without explicitly mentioning the mental states that make these reasons available to the agent.

Another kind of additional complexity arises from the possibility of error. We sometimes (intentionally) do something, despite having no good reason for doing it. You may go to the cafeteria because you mistakenly think it is still open, or because you mistakenly take it to be desirable to eat three full meals a day, despite your doctor’s orders to the contrary. In such cases, we resort to *explanantia* that are neutral on whether you have a good reason for your action: we appeal to what you believe, or find desirable, or want. There is a sense, though, in which our grasp of such ‘neutral’ explanations depends on our grasp of explanations in terms of normative reasons. Understanding the explanatory connection between what you (merely) believe or find desirable and your intentional actions requires understanding that *if* your attitudes were correct and knowledgeable, this would give you a good reason to perform those actions. It also requires seeing you as an agent who is *able* to act for normative reasons (even if, on a particular occasion, your action may reflect some misapprehension). The explanatory force of non-factive attitudes (as regards intentional actions) cannot be understood in isolation, independently of our grasp of what is involved in acting for normative reasons (Hornsby 2008; Roessler and Perner 2013; Roessler 2014). We want to suggest that ‘teleology-in-perspective’ differs significantly from the three most influential views of the ‘folk psychology’ of intentional action (theory theory, simulation theory, and rationality theory), and that it has significant advantages over its rivals.

## 2 Competing theories

### 2.1 Theory theory

The prevalent suggestion of how we understand and predict actions is by way of a theory of mind, in which mental states play the role of theoretical terms. They cannot be observed but must be inferred from observable facts (Sellars 1956; Premack and Woodruff 1978; Churchland 1984). Actions are defined as movements caused by a mental event, the content of which relates appropriately to the performance of the action (Davidson 1963; Brand 1984). In this tradition, in particular in developmental work, this theory of mind is seen as a primarily causal theory of abstracted regularities. Given certain observable conditions, one can infer certain mental states, from which one can infer what the agent is likely to do: for example, if I hear your stomach groan I infer that you want to get food (desire) and, assuming you know (believe) that you can get some in the cafeteria, I can use a basic principle of theory of mind for action prediction. In the words of Gopnik and Meltzoff (1997, 126):

[The main tenets of the adult theory of mind] are perhaps best summarized by the ‘practical syllogism:’ ‘If a psychological agent wants event y and believes that action x will cause event y, he will do x.’ Many philosophers have argued that the practical syllogism is the basic explanatory schema of folk psychology.

The ‘practical syllogism’ recalls the means-ends structure of practical reasoning in teleology, where information about a desired state is combined with information about how to achieve that state. There are, however, some critical differences.

(1) *Mental states*. Gopnik and Meltzoff’s ‘practical syllogism’ employs mental states that are directed at states of the world, while a teleologist is concerned primarily with worldly facts. The teleologist considers not simply a desire for getting food but, primarily, the desirability of getting food and, instead of looking at the agent’s beliefs about how to achieve this, she first of all asks whether the agent knows how to achieve the desirable state.
(2) *Reasons versus regularities*. The ‘practical syllogism’ in Gopnik and Meltzoff’s hands is simply a lawlike generalization. It captures a certain regularity in people’s behaviour, comparable with lawlike generalizations concerning the way inanimate objects work. There is no mention of normative reasons, nor any implication that the agents whose actions are intelligible in terms of the ‘practical syllogism’ act for what they themselves take to be good reasons. Thus the theory theory cannot respect the intuition that intentional actions are intelligible in terms of the agent’s reasons, rather than just as instances of lawlike generalizations. (It is significant here that Gopnik and Meltzoff’s terminology is infelicitous; a ‘practical syllogism’, as understood in the Aristotelian tradition (Anscombe 1957), is a type of inference used in *practical reasoning*, not a lawlike generalization.)
(3) *Values*. Reasons require value judgements. Unless there is something good about a state of the world, there can be no good reason for bringing about that state; it cannot be an intelligible goal for an action.
(4) *Normativity*. Teleology sees people as, in the paradigmatic, basic case, acting for good reasons. (Acting on the basis of error or confusion is a not fully successful attempt to exercise the capacity to act for good reasons.) Since the reasons are judged to be good, one has the sense that one ought/should do what one has good reasons to do. This gives actions a normative and rational dimension lacking in theory theory. The latter sees people as pursuing whatever they desire. The normative question of whether an action *makes sense* is deemed completely irrelevant in the context of actions explanation. For the teleologist, that is the first question we need to ask in order to understand an action as intentional. If it turns out the action makes no sense, we must either invoke the way it may *appear* to make sense from the agent’s point of view, or we have to abandon the attempt to explain it as an intentional action.

In sum, we think that on these dimensions of divergence teleology is closer to the pulse of common sense than theory theory. It captures our intuitive view of the other much better.

### 2.2 Simulation theory

Understanding the other by simulation consists of imagining having the other’s first-person view of his situation (imaginative identification). This limits simulation to sentient beings (for a deeper discussion of this issue, see Engelen (2018)). Although only imagined, the situation nevertheless triggers pretend versions of mental processes similar to the mental processes triggered by the real situation in the other. By introspection (Goldman 2006; or by ascent routines, Gordon 1995, 2007) of these pretend states, one can know about the actual mental states of the other. If I hear your stomach growl I imagine being hungry, which triggers mental pretend states in me, including the pretend intention to visit the cafeteria. I attribute this intention to you in real life.

Mental simulation, thus, shares several features of teleology. It makes the other intelligible as acting for good reasons, subject to reasons’ normative force, and being rational. There are also clear differences between teleology and simulation theory:

(1) *Objectivity and pretense*. The teleologist deals only in objective facts (as they present themselves to the teleologist) processed by her mind, whereas the simulationist deals with imagined facts processed in pretend mode. Walking through a dark park imagining being followed by a devious-looking character plausibly elicits similar emotions and a similar tendency to quicken one’s pace as one might do when actually being followed by such a character. When this happens, we are aware that our feelings are caused by the imagination and not reality. Although one can use this as a technique to figure out what goes on in the other, it seems unlikely that we do so on a regular basis and not be aware that we do so (see Gallagher 2007).
(2) *Privacy*. From the objectivity of practical reasons, it follows that they are also public (for anyone in the right place to perceive them; Roessler and Perner 2015). Of course, I may not know some relevant facts about you, for example, that you are hungry and therefore have a reason to go to the cafeteria. But once I know of your hunger I can see it as a reason for you to go to the cafeteria. Simulation theory sees the reason-giving feature of your hunger as your private fact for you, which can only be revealed to me (and others) by simulating you.
(3) *Introspection*. With simulation one needs to introspect^1^ on one’s own mental states before one can attribute them to the other. There is ongoing theoretical controversy about the possibility of introspection (e.g. Carruthers 2011), which makes it advantageous for teleology to be able to do without.
(4) *Perspective.* Theory theory takes a third-person perspective on the other’s mind, while simulation theory affords the other’s simulated first-person view. But what type of perspective does teleology afford? The teleologist takes a third-person view of the situation the other is in. She does not imagine being in the other’s situation. However, she needs to engage in the same evaluation of this situation and what needs doing to improve the situation as the other does, that is, the practical reasoning has to be essentially shared. Indeed, there is a sense in which teleology has a second-person flavour: a successful answer to the second-person question ‘Why are you doing X?’ would precisely enable the questioner to find your doing X intelligible by recognizing and appreciating your normative reasons for doing X (Roessler 2014) (on the importance of the second-person perspective in development, see the contribution by Musholt (2018)). So the best characterization, for the moment, is that teleological reasoning is not tied to any particular perspective.

### 2.3 Rationality theory

Goldman (2006) lists as the third major approach to understanding the other the so-called Rationality or Charity Theory (Davidson 1963): ‘Attributors decide what attitudes to ascribe to others by crediting them with rationality and determining what rationality dictates in their circumstances’ (Goldman 2006, 23). In relation to understanding intentional action, the idea is this. We find intentional actions intelligible by seeing agents as conforming to the basic ‘requirement of instrumental rationality,’ which, very roughly, says that if you want to bring about x and believe that doing y is the most effective way to do so, then you ought to do y.

It may seem as if rationality theory effectively met our earlier criticism of theory theory, of failing to give any role to the normative question of whether an action ‘makes sense.’ The trouble is that the operative notion of ‘making sense’ is far removed from the way we ordinarily think about what people ought to do. Actions are seen as ‘making sense’ not in the light of the features that make them desirable (e.g. an action’s being fun or required to alleviate someone’s distress or conducive to one’s well-being) but by reference to a certain abstract and completely general demand of something called rationality. There are two central problems here. One is that it is far from clear how the idea is to be articulated. If your instrumental belief that you can bring about x by doing y is false, there is a straightforward sense in which it is not true that you ought to do y. (It would, for example, be highly misleading for us to advise you that you ought to do y, given our background knowledge that your belief is false.) The rationality theory is apparently committed to insisting that there must nevertheless also be a sense in which it is true that you ought to do y – given your desire to bring about x, and your (albeit false) belief. It is not clear if the idea of a distinction between the ‘ought of reasons’ and the ‘ought of rationality’ can be sustained. The other problem has to do with the question of *why* we ought to conform to the ‘demands of rationality.’ Philosophers have produced theories as to what gives us a good reason to ‘be rational’ (e.g. Bratman 2009), but these are philosophers’ reasons, not considerations available to common-sense psychology. Rationality theory’s analysis of our ordinary explanatory practice gives a central role to normative demands the rationale of which is, to common-sense psychology, at best elusive.

Teleology can avoid these problems. It conceives of rationality not in terms of a system of abstract requirements governing the internal relations among our attitudes, but in terms of our capacity to act for normative reasons. In Josef Raz’s words: ‘An account of rationality is an account of the capacity to perceive reasons and to conform to them’ (1999, 355). Teleology’s rationality takes it that what you ought to do depends on what you have reason to do. No principles of rationality have to be formulated a priori.

Csibra and Gergely (1998) proposed in the tradition of rationality theory that infants from around nine months understand animate action within a ‘teleological’ scheme of movements that adjust to given circumstances in order to reach a goal. Although they use the word ‘reason’ and although they refer to ‘constraints of reality’ rather than to the agent’s attitudes, the normative element in their ‘teleological schema’ derives from a putative ‘rational requirement,’ that one ought to take the optimal means to reach one’s goals, as the following passage brings out:

reference to a future state is accepted as a teleological explanation (reason) for a behaviour in case it justifies it, i.e. when, given the constraints of reality, the behaviour can be seen as a rational way to bring about the goal state. (Csibra and Gergely 1998, 256)

This contrasts with our approach where reasons come first and conformity to reasons makes actions rational. Furthermore, good reasons depend on something worthwhile (the goal) to be pursued. Hence the absence of any mention of values in Csibra and Gergely’s ‘rational’ teleology highlights the difference to our ‘reason-based’ (or Aristotelian) teleology, for which value judgements are essential.

### 2.4 Comparison

Table 1 provides an overview of how teleology compares to its competitors on the different dimensions of the above discussion. We marked each theory with a check mark if its position on a dimension conforms to (our) intuition, and with a cross otherwise. As one can see, teleology comes out with all favourable features but one great problem. It cannot account for the rationality of actions based on mistaken beliefs or values that diverge from one’s own because it has no way to adjust to different perspectives (last row of Table 1). We turn to the question of how to amend this shortcoming in Section 4 after some preparatory comments in Section 3.

## 3 Competency

The core tenet of teleology is that people act for good reasons. This informs about what people should or ought to do. A further assumption for predicting actual action is that people will do what they ought to do. But clearly people cannot be expected to do everything for which they have some reason to act. How can we constrain the field of worth-while actions to those that are likely to occur? Perner and Esken (2015) subsumed the needed constraints under *competency*, a particularly useful term as it covers the ability to carry out as well as the societal responsibility for taking on the needed action. For example, the fact that you are hungry gives you good reason to get some food in the cafeteria, and I can predict that you will, therefore, go there. However, if you had a recent knee operation and can’t walk you won’t be able (competent) to do so. Moreover, your hunger and your inability also give me reason to go to the cafeteria and get some food for you.

A particularly relevant element of competency is informational access. Only a person who has access to the reason-giving facts is competent (able) to act on these reasons. For vision, this means that only an event that takes place within the person’s visual field gives her reason to act on the fact that this event has taken place. Although this could be construed as involving a mental state, that is, the person *seeing* and therefore *knowing* of the event, one can simply see it as a pure fact that something took place at a certain place defined by the person’s visual field. This allows the teleologist to understand the rudiments of communication without the ability to comprehend mental states (Perner and Esken 2015). A pointing gesture is an action that enables the recipient to direct his eyes (attend) to a relevant event or state, which provides a reason for action. However, registration of reason-giving facts also provides the launch pad for understanding other people’s different perspectives, which helps overcome teleology’s central impediment.

## 4 Teleology in perspective

Table 1 shows that teleology has much to recommend itself but for one serious shortcoming. It has no way to accommodate rational behaviour when the other does not share the teleologist’s own view of the facts and values. A classic test of children’s ability to adjust to different perspectives is the false belief test (Wimmer and Perner 1983): Mistaken Max did not witness the unexpected transfer of his chocolate to a new location and thus mistakenly thinks it is still in its original location. Children are asked where Max will look for his chocolate. Children before the age of about four years (Wellman, Cross, and Watson 2001) answer with the chocolate’s actual location as a teleologist would, since the objective facts give Max reason to go to that location. The older children answer as an adult would: Max will look in the old location. What changes around age four? Are children shedding teleology in favour of a new approach, a belief-desire theory, or do they amend their familiar teleology in a way that makes it applicable to such error cases?

Perner and Roessler (2010) suggested that children become able to use their teleology within the other’s perspective and called it ‘teleology-in-perspective’ (on the role of perspective taking in social conflicts see the contributions by Buddeberg (2018) and Breithaupt (2018)). This would enable older children as well as adults to maintain the intuitive advantages of teleology. However, this suggestion raises two fundamental questions: (1) How does one know what another person’s perspective is? Wouldn’t one have to use a theory or simulation to figure that out? (2) How can one use practical reasoning based on objective facts within another person’s perspective populated with subjective ‘facts’?

### 4.1 How to get the other’s perspective

Indeed, one could adopt suggestions from theory theory or simulation to explain how we can know another person’s beliefs that define her perspective. The approach sketched here combines theoretical and simulative intuitions and is limited to the typical test paradigms assessing children’s competence. As argued above, for a fact to become a reason for action it is necessary that the relevant event takes place in the informational field (e.g. visual field or analogous construct for other senses and linguistic information) so that the fact that this event took place can be registered by the agent (Apperly and Butterfill 2009) and put into one’s experiential record (Perner and Roessler 2010, 2012) for that person. This experiential record defines the other’s perspective. So, in Mistaken Max’s case, this means that the chocolate’s move to the new location does not appear in one’s experiential record for Max. Hence it is not part of his perspective. Notice, we can establish Max’s perspective without imaginative identification with Max as in simulation. Registration of objective facts about events in Max’s informational field suffices.

### 4.2 Practical reasoning with the other’s perspective

How should the teleologist now proceed with practical reasoning from Max’s experiential record to the insight that Max has good reasons to believe that his chocolate is still in its original place and to go there for it? The objective fact of the chocolate being in its new place does not provide good reasons for this belief and action. Perner and Roessler (2010; Roessler and Perner 2013) suggested that the teleologist could reason counterfactually by taking the experiential record for Max as a counterfactual antecedent: ‘If the chocolate were still in its original location then there would be good reasons to believe it was still there and to go there to get it.’ Importantly, the counterfactual reasoning preserves the normative aspect of Max’s action within the counterfactual world, which tends to be lost in theory theory. This has some affinity with simulation as one stipulates a counterfactual antecedent that corresponds to Max’s viewpoint, draws a counterfactual conclusion, and then ascribes the result to Max as what he has good reasons to believe and how to act. Nevertheless, our proposal falls short of having to engage in imaginative identification with Max, seeing events through his eyes.

Presumably, to get theory theory off the ground similar objective facts as in one’s experiential record need to be detected in order to apply knowledge like ‘if Max didn’t see the chocolate moved he will think it is still in its old location and he will go there when looking for his chocolate.’ But this is not practical reasoning engaging one’s own mind about the subject matter that Max is concerned with. It is reasoning about Max. It should also be noted that there is no need for counterfactual reasoning. The fact that Max is not seeing the chocolate’s transfer leads to the fact that he believes it to be in its old location, and so forth. Counterfactual reasoning, ‘if Max had seen the transfer … ’ is simply not applicable.

The important conclusion here is that teleology-in-perspective requires children to reason counterfactually. Hence children should not be able to know what another person falsely believes and how this person will act before they can reason counterfactually. A prediction neither theory theory nor simulation theory make. We will shortly take up the relevant empirical evidence.

## 5 Development

Empirical evidence for our suggestions comes mainly from developmental psychology. Teleology can make sense of the known progression of how children understand actions. Perner and Esken (2015, 77–79 and 83–85) give a succinct overview. Here we concentrate on three pieces of evidence in favour of teleology and against competing theories.

### 5.1 Competition

Priewasser, Roessler, and Perner (2013) tested the prediction (Perner and Roessler 2010) that children are limited to basic teleology until about four years, when they become able to understand perspective differences and use teleology-in-perspective. The standard theory-theory position holds that children first develop the concept of desire and only around four years the concept of belief (Wellman 1990: from a desire to a belief-desire psychology). According to teleology, goals (relating to desire) and means to achieve the goal (relating to belief) should be mastered well before four years. Only when a difference of perspective becomes relevant are tasks mastered later. A difference in perspective about the best means to achieve a goal is assessed by the well-known false belief test around four years. Teleology, but not Wellman’s theory, implies that a difference in perspective of action goals should be understood equally late.

**Figure 1:**
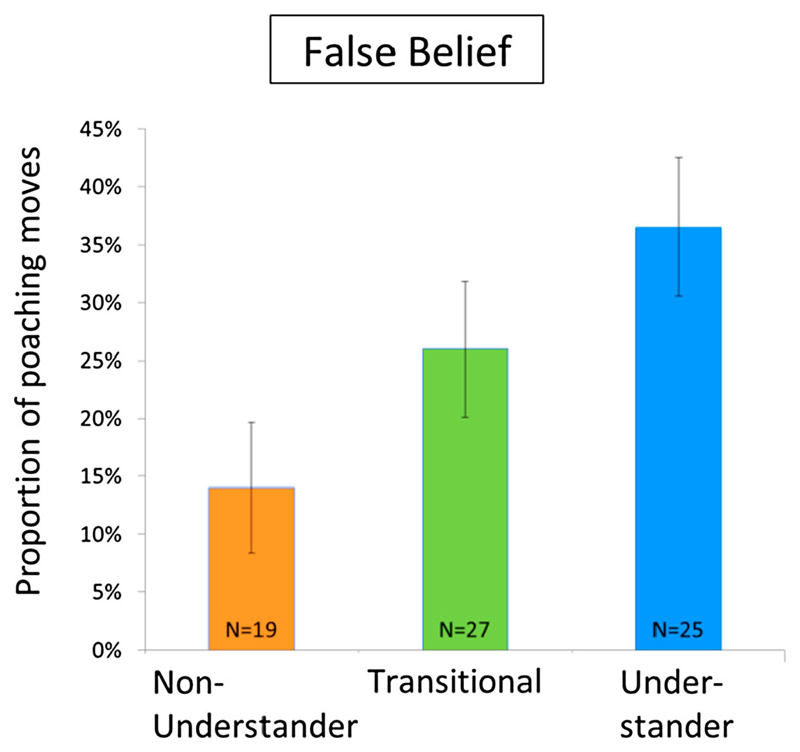
Children’s number of competitive poaching moves in relation to their passing the false belief test. Data from Priewasser, Roessler, and Perner (2013).

Priewasser, Roessler, and Perner (2013) used a competitive bead-collecting game to test this. Sessions consisted of three children taking turns in throwing dice to collect more beads to fill a stack. Whoever finishes her stack first, wins. Importantly, children were explicitly told that one could take the beads either from a common source or poach from another player. Teleologists should have problems appreciating the point of poaching moves, because action goals must be objectively desirable and the goal to diminish the other’s stack and the goal to finish that stack cannot both be desirable. Only when children are able to understand that players take different perspectives on what is ‘desirable’ do the competitive poaching moves make sense. Consequently, children should engage in competitive thwarting moves like poaching only when they pass the false belief test as an index of their perspective-taking ability. This is what happened, as shown in Figure 1. Children who passed the false belief test made two-and-a-half times more poaching moves than those who failed the belief test (on the dark side of empathy, see Breithaupt (2018)).

### 5.2 Pragmatic cues

**Figure 2:**
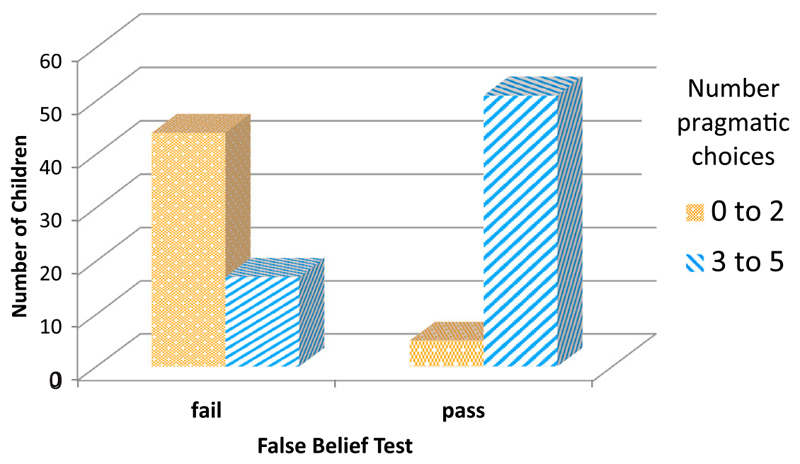
Number of children with a majority of pragmatically adequate choices overcoming the mutual exclusivity bias. Data from Gollek and Doherty (2016, Experiments 2 and 3).

Haryu and Imai (1999) assessed when children take pragmatic information about a speaker’s desire into account for resolving reference in the context of the well-known mutual exclusivity bias. Faced with a familiar object, for example, an apple, and a strange new, non-edible object, most three- to five-year-olds, when asked to hand a puppet the *heku* (a nonsense word in Japanese), hand the puppet the novel object. In another condition, these children were told beforehand that the puppet was very hungry – a strong pragmatic cue to provide something edible. The younger children still handed the inedible novel object, while most five-year-olds gave the puppet the apple. This effect fits nicely the transition from teleology to teleology-in-perspective. The question is why the young teleologists have a problem with this task. An answer is provided by the view that different names create different perspectives (Clark 1997; Tomasello 1999), that is, ‘apple’ and ‘heku.’ Children, still incapable of teleology-in-perspective, cannot understand such duality of perspectives: once they see it as an *apple*, they can’t see it as a *heku* at the same time. Only older children, who pass the false belief test, can override this constraint. Indeed, Gollek and Doherty (2016) found (Figure 2) that only children who passed the belief test preferred to hand the puppet the apple rather than the inedible, unnamed object.

### 5.3 Counterfactual reasoning and understanding belief

Practical reasoning within another perspective can be done by the first-person identification technique of simulation theory. Alternatively, we suggested, one can use counterfactual reasoning. Whatever the other experiences (or was told) serves as the counterfactual antecedent. For example, Mistaken Max did not witness the transfer of his chocolate to the new location, so his experiential record shows his chocolate as being in its original location. Hence the teleologist reasons: ‘If Max’s chocolate were still in its original place Max would have good reason to (a) believe it is still there and (b) go to that place to get it.’ If this is how we and children do it then children’s ability to make correct belief and action predictions should depend on their level of counterfactual reasoning.

Such a relationship has been reported at the age around four years by Riggs et al. (1998). Rafetseder, Cristi-Vargas, and Perner (2010, Rafetseder, Schwitalla, and Perner 2013) found that some counterfactual tasks are far beyond the reach of four-year-olds’ reasoning ability and are mastered not before seven years or later. For instance, seven- to eight-year-old children were told how things work in a particular household:

When mother buys sweets she puts them either on the upper or lower shelf. Sometimes her tall son comes and takes the sweets to his room. Sometimes her small daughter comes and takes them to her room but only when they are on the lower shelf, because she can’t reach the upper shelf.

An easy story within this framework was: ‘Mother puts sweets on the upper shelf and the girl comes to get them. Where are the sweets now?’ Almost all children answered correctly with the upper shelf. They also answered the counterfactual question: ‘If not the girl but the boy had come to look for sweets, where would they be?’ correctly with ‘boy’s room.’ Now for a hard version: ‘Mother puts sweets on the upper shelf and the boy comes to get them. Where are the sweets now?’ Again mostly correct answers: ‘boy’s room.’ But the counterfactual question: ‘If not the boy but the girl had come to look for sweets, where would they be?’ was now answered by most children incorrectly with ‘girl’s room,’ instead of ‘top shelf.’ Only by about 14 years did most children answer correctly.

**Figure 3:**
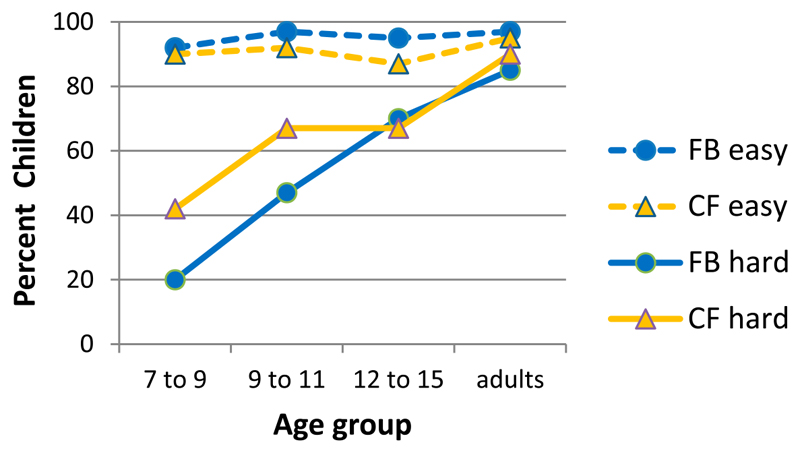
Per cent children who give the correct answer to counterfactual and corresponding false belief questions. Data from Leahy, Rafetseder, and Perner (2017).

Leahy, Rafetseder, and Perner (2017) amended the first, easy story with mother mistaking the girl for the boy because the girl was wearing the boy’s jacket. For the second, difficult story mother thought the boy was the girl as he stooped when returning to his room. With these amendments it was possible to ask a counterfactual question as well as a matched false belief question: ‘Where does mother think the sweets are now?’ Teleology is committed to predicting that children’s answers parallel their answers to the counterfactual question. This means children should answer correctly with ‘on the upper shelf’ in the first but incorrectly with ‘girl’s room’ in the second story. This is what happened as shown in Figure 3.

It is difficult to see how this result can be made to fit theory or simulation theory without making specific amendments. For instance, if children took mother’s first-person perspective to figure out their answer to the belief question, then there could be no relationship with the counterfactual question since mother does not engage in counterfactual reasoning. Simulating mother in story 2: ‘I (mother) put the sweets on the top shelf. My girl came to look for them. She can’t reach the top shelf. So they are still there.’ Such a simulation of mother should provide children with the correct answer ‘on the top shelf’ and not ‘in the girl’s room.’

## 6 Conclusion

We have tried to present persuasive arguments why teleology captures our common-sense way of understanding the other’s intentional actions better than competitor theories. It captures that we consider other people to act in essential ways for the same reason as we do without having to deploy special techniques like imaginative identification on a routine basis. We have also presented three pieces of recent developmental evidence that confirm predictions from teleology-in-perspective as the way in which teleology can be applied to people’s differing perspectives.

## Figures and Tables

**Table 1:** Comparison of four theories of how we understand the other.

Dimension	Theory			
	Theory	Simulation	Rationality	Teleology
Special domain	no 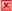	yes 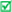	yes-but 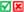	yes 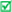
Reasons	no 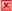	private 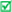	yes-but 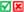	objective 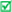
Perspective	3^rd^ 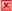	1^st^ 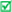	3^rd^ 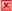	no paticular ?
Introspection	no 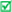	yes 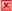	no 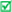	no 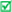
Pretend states	no 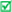	yes 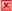	no 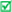	no 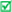
Subjectivity	private 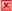	private 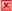	public 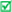	public 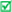
Perspective difference	yes 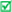	yes 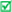	no 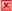	no 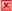

## Note

1 This is only required in Goldman’s (2006) version. Gordon (1995) suggested replacing introspection with ascent routines (Evans 1982). Whether this is possible for all mental states is still controversial (Barlassina and Gordon 2017, Section 6.2).
